# “I have brain fog…” About cognitive impairment after COVID-19

**DOI:** 10.1192/j.eurpsy.2022.1292

**Published:** 2022-09-01

**Authors:** H. Ghabi, A. Aissa, A. Zili, A. Ben Cheikh Ahmed, Y. Zgueb, S. Madouri, U. Ouali, R. Jomli

**Affiliations:** 1 Razi Hospital, Department Of Psychiatry A, manouba, Tunisia; 2 Razi Hospital, Department Of Psychiatry A, Manouba, Tunisia

**Keywords:** Covid-19, cognitive impairment

## Abstract

**Introduction:**

Much has been learned about SARS-CoV-2. However, the mechanism of short or long-term neuropsychiatric symptoms remains unclear. several hypotheses, including lack of oxygen caused by lung damage, inflammation affecting brain cells, or Lack of blood flow caused by swelling of the small blood vessels in the brain, have been advanced to explain these symptoms.

**Objectives:**

Herein, we presented a case of cognitive impairment diagnosed after infection with COVID 19.

**Methods:**

We discussed, through a clinical case, the possible mechanisms and risk factors of cognitive impairment following COVID 19 infection.

**Results:**

This case concerned a 28 –year-old patient. He had no personal or family psychiatric. In August 2021, he presented a SARS-CoV-2 infection without hypoxemia or respiratory failure. On day 10 the patient recovered. Two days after, he consulted our psychiatric department as he experienced impairment in memory. He had impairment in attention and executive function, and in particular verbal fluency. He said that his thinking was sluggish, fuzzy, and not sharp. *He denied any* alcohol or *drug abuse.* He was euthymic and he had no depressive symptoms. Arterial blood gas, laboratory, and clinical findings were normal. A brain CT scan with contrast was performed and did not show any abnormality.

**Conclusions:**

This case highlighted the possible cognitive consequences of COVID-19 during the recovery phase. Further work is required to identify risk factors of psychiatric symptoms following COVID-19 infection and their management.

**Disclosure:**

No significant relationships.

